# Sclerosing encapsulating peritonitis in advanced liver cirrhosis presenting with acute small bowel obstruction: a case report and literature review

**DOI:** 10.1093/jscr/rjaf159

**Published:** 2025-03-26

**Authors:** Louis Britten-Jones, Juanita Chui, Michael Yulong Wu, Geoffrey Chu, Robert Knox, Ruwanthi Wijayawardana

**Affiliations:** Department of General Surgery, Orange Health Service, 1530 Forest Rd, Orange NSW 2800, Australia; Department of General Surgery, Orange Health Service, 1530 Forest Rd, Orange NSW 2800, Australia; Faculty of Medical and Health Sciences, The University of Sydney, NSW 2006, Australia; Department of General Surgery, Orange Health Service, 1530 Forest Rd, Orange NSW 2800, Australia; Faculty of Medical and Health Sciences, The University of Sydney, NSW 2006, Australia; Faculty of Medical and Health Sciences, The University of Sydney, NSW 2006, Australia; Department of Gastroenterology, Orange Health Service, 1530 Forest Rd, Orange NSW 2800, Australia; Department of General Surgery, Orange Health Service, 1530 Forest Rd, Orange NSW 2800, Australia; Faculty of Medical and Health Sciences, The University of Sydney, NSW 2006, Australia; Department of General Surgery, Orange Health Service, 1530 Forest Rd, Orange NSW 2800, Australia; Faculty of Medical and Health Sciences, The University of Sydney, NSW 2006, Australia

**Keywords:** sclerosing encapsulating peritonitis, abdominal cocoon syndrome, small bowel obstruction, liver cirrhosis

## Abstract

Sclerosing encapsulating peritonitis (SEP) is an inflammatory condition characterized by the encasement of the small bowel in a dense fibro-collagenous membrane resulting in acute bowel obstruction. In the context of liver disease, SEP is exceptionally rare, with only eight known cases to date. We describe the case of a 52-year-old male presenting to a rural hospital with acute small bowel obstruction secondary to SEP in the setting of decompensated liver cirrhosis. SEP is an uncommon cause of acute small bowel obstruction. Definitive treatment requires surgical intervention, typically with enterolysis and excision of the abdominal cocoon. For patients with cirrhosis, this is associated with exceptionally high perioperative morbidity and mortality. SEP is an important differential in cirrhotic patients presenting with acute bowel obstruction, particularly in the context of long-term beta blocker use. Early discussions regarding prognosis and careful patient selection for surgical intervention are of paramount importance.

## Introduction

Sclerosing encapsulating peritonitis (SEP), also known as abdominal cocoon syndrome, is a rare inflammatory condition characterized by the encasement of the small bowel in a dense fibro-collagenous membrane, leading to symptoms of small bowel obstruction. Although the aetiology of SEP is not fully understood, it may be idiopathic or secondary to an underlying cause of peritoneal inflammation [1]. It is most often seen in the setting of peritoneal dialysis (PD) and recurrent peritoneal infections with an estimated incidence of 0.3%–3.9% [2]. Here, we describe a case of acute small bowel obstruction secondary to SEP in a patient with decompensated liver cirrhosis. The sparse literature on this condition is summarized, highlighting the challenges associated with diagnosis and management.

## Case report

A 52-year-old male presented to a rural Australian hospital with acute confusion, abdominal pain, and intractable vomiting. This was on a background of Child-Pugh C alcoholic liver cirrhosis with portal hypertension and previous ascites requiring large-volume paracentesis. He had been on long-term beta-blockers (Carvedilol 12.5 mg BD) for primary prophylaxis of variceal bleeding. Other relevant past medical history included anxiety and depression. There was no history of abdominal surgery. On examination, the patient was jaundiced and encephalopathic. His abdomen was distended with a palpable firm central mass. Investigations showed elevated inflammatory markers (white cell count 13.2 × 10^9^/l, C-reactive protein 125 mg/l), acute kidney injury (creatinine 161 μmol/l), and cholestatic liver enzymes (alanine aminotransferase 29 U/l, aspartate aminotransferase 44 U/l, gamma-glutamyl transpeptidase 265 U/l, and alkaline phosphatase 303 U/l). Elevated total bilirubin 65 μmol/l and low serum albumin 20 g/l suggested hepatic decompensation, however, INR 1.0 was normal. Normal platelet count 188 × 10^9^/l. At the time of presentation, the patient’s cirrhosis mortality scores were Child-Pugh C-11 and Model for End-Stage Liver Disease (MELD-Na) 21.

**Figure 1:**
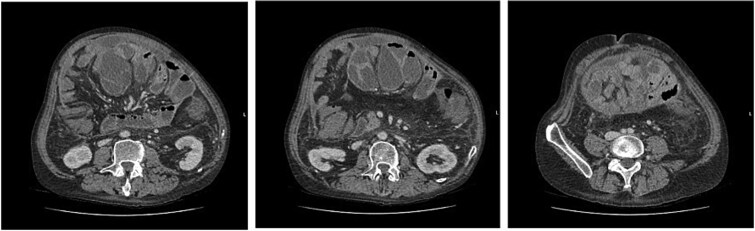
(left to right–cranial to caudal) Axial contrast-enhanced CT images demonstrating dilated small bowel loops in the central abdomen, encapsulated in thickened peritoneum and with trace free fluid. Appearances are consistent with small bowel obstruction.

Abdominal computed tomography (CT) scan revealed multiple dilated small bowel loops with air-fluid levels, encapsulated within thickened peritoneum. There were collapsed bowel loops distally, consistent with an acute small bowel obstruction (Fig. 1). This was associated with trace-free fluid and no features of ischaemic bowel or pneumoperitoneum. A radiological diagnosis of SEP was made.

Given the patient’s decompensated liver cirrhosis and high surgical risk, the decision was made to trial conservative management in the first instance. The patient was managed with nasogastric decompression and kept nil-by-mouth. His hepatic encephalopathy was managed with titration of regular lactulose, and rifaximin was commenced as his bowel obstruction began to resolve. By the sixth day of his admission, he was tolerating a regular diet, his bowels were opening, and he was deemed safe for discharge. Unfortunately, the patient developed recurrent symptoms within 48 hours. On repeat presentation, he was tachycardic, tachypnoeic, and had generalized peritonism on examination. Venous blood gas demonstrated a lactate of 5.7 mmol/l. Bloods demonstrated CRP of 100 mg/l and WCC of 18 × 10^9^/l. A repeat CT scan was concerning for recurrent small bowel obstruction. There was clinical concern for evolving ischemia from hypoperfusion of the encapsulated small bowel. The patient was referred to a quaternary centre with a specialist liver transplant unit on this occasion. Following multidisciplinary team and family discussions, the decision was made for non-operative management due to high estimated perioperative mortality, ongoing alcohol use precluding liver transplantation in the event of post-operative hepatic decompensation, and the guarded prognosis associated with this condition. The patient was transitioned to comfort-based care and died 7 days later.

## Discussion

Sclerosing encapsulating peritonitis (SEP) is an uncommon condition that is associated with high morbidity and mortality risk. It typically occurs in comorbid patients with a history of long-term PD, sarcoidosis, peritoneal malignancy, and infection, such as peritoneal tuberculosis [1]. Although not fully understood, a “two-hit” hypothesis has been proposed, where an inflammatory stimulus in addition to non-inflammatory peritoneal sclerosis leads to the development of SEP [3].

Rarely, liver cirrhosis has been associated with SEP [4–10]. This has been described in eight reports to date (Table 1). Some studies have identified beta-blockers as an independent risk factor for the development of SEP [7–15]. For patients with cirrhosis, beta-blocker use has now become the standard of care for secondary variceal bleeding prophylaxis [16, 17] and clinicians should be aware of the risk for SEP.

**Table 1:** Summary of the reported cases of SEP in the context of liver cirrhosis in the literature to date

**Study**	**Demographics**	**Presentation**	**Preoperative investigations**	**Beta-blocker use (agent and duration)**	**Treatment of SEP (conservative vs laparotomy) and outcome**
Seng *et al.* 1993 [4]	17 M liver cirrhosis of unspecified aetiology	Recurrent small bowel obstruction	Abdominal CT	None	Laparotomy. Discharged
Yamamoto *et al.* 2004 [5]	57 M chronic hepatitis B	Hepatic encephalopathy	Diagnosed at time of liver transplantation	Not reported	Unable to proceed with liver transplantation. No further surgical intervention. Died 17 days later of liver failure
Yamamoto *et al.* 2004 [5]	52 M alcoholic liver cirrhosis	Recurrent small bowel obstruction	Abdominal CT	Not reported	Laparotomy—excision of abdominal cocoon and total enterolysis discharged (free of symptoms 15 months later)
Wakabayashi *et al.* 2007 [6]	37 M alcoholic liver cirrhosis	Recurrent small bowel obstruction Perforated peritonitis	Abdominal CT	Unknown	Laparotomy—adhesiolysis and small bowel resection. Discharged
de Vos tot Nederveen Cappel *et al.* 2008 [7]	65 M alpha-1 antitrypsin deficiency	Fatigue	Diagnosed at time of liver transplantation	Propranolol 6 years	Laparotomy—unable to proceed with liver transplantation; deferred. Subsequent successful transplantation following further investigations to exclude malignancy and histopathology confirmation of SEP. Discharged
Kalra *et al.* 2009 [8]	59 M cryptogenic cirrhosis of the liver and portal hypertension	Recurrent small bowel obstruction	Abdominal CT	Propranolol 4 years	Laparotomy—extensive adhesiolysis. Discharged
Noh *et al.* 2016 [9]	46 M Chronic hepatitis B	Recurrent small bowel obstruction	Abdominal CT	Propranolol 15 years	Laparotomy—excision of abdominal cocoon. Discharged
Asotibe *et al.* 2020 [10]	64 M Child-Pugh B liver cirrhosis of unspecified aetiology	Recurrent small bowel obstruction	Abdominal CT	Propranolol 3 years	Laparotomy—excision of abdominal cocoon and extensive adhesiolysis. Discharged

The diagnosis of SEP can be challenging in the absence of obvious radiologic features. SEP is a slow-developing disease and often not identified until patients become symptomatic from the progressive fibrotic encasement of the small bowel. From previous reports, some patients are not diagnosed until laparotomy [18].

There is presently no consensus on the approach to management of this rare condition. When identified at an early stage, there is limited evidence to support the efficacy of immunosuppressive agents or selective oestrogen receptor modifiers with antifibrotic properties, such as tamoxifen [1]. Definitive treatment requires surgical intervention, typically with enterolysis and excision of the abdominal cocoon. Unfortunately, acute small bowel obstruction associated with SEP carries a high mortality risk. Existing reports on this condition have predominantly been single-centre series in the context of PD. The largest cohort study on surgical treatment of SEP diagnosed radiologically reports a mortality rate of 25.1% in this patient cohort [19], while mortality rates associated with acute presentations have been as high as 76.9% [20]. For cirrhosis patients, the perioperative morbidity and mortality associated with this condition is poorly defined but is expected to be high when there is hepatic decompensation. From the scarce literature, publication bias needs to be considered. Furthermore, patients presenting with SEP tend to have significant systemic comorbidity. Of note, almost all reported cases on SEP in the context of liver cirrhosis proceeded to laparotomy. These patients were noted to be haemodynamically stable with compensated liver disease.

## Conclusion

This case contributes to the limited literature on SEP in liver cirrhosis, recognizing the routine use of beta-blockers for primary prophylaxis of variceal bleeding as a potential risk factor. This report highlights the challenges in managing acute small bowel obstruction secondary to SEP in this high-risk cohort. Early referral to a liver transplant unit should be made on recognition of this condition, and surgical candidates should be offered timely definitive treatment. Appropriate patient selection is paramount. Given the high risk of mortality with SEP, early discussions surrounding prognosis and the option for palliative care are recommended in all patients.
